# *catena*-Poly[[(4,4′-dimethyl-2,2′-bipyridine-κ^2^ *N*,*N*′)cadmium]-di-μ-bromido]

**DOI:** 10.1107/S1600536812046636

**Published:** 2012-11-17

**Authors:** Sadif A. Shirvan, Sara Haydari Dezfuli, Fereydoon Khazali, Ali Borsalani

**Affiliations:** a Department of Chemistry, Omidieh Branch, Islamic Azad University, Omidieh, Iran; b Department of Petroleum Engineering, Omidieh Branch, Islamic Azad University, Omidieh, Iran

## Abstract

In the crystal of the title polymeric compound, [CdBr_2_(C_12_H_12_N_2_)]_*n*_, the Cd^II^ cation is located on a twofold rotation axis and is six-coordinated in a distorted octa­hedral geometry formed by two N atoms from the 4,4′-dimethyl-2,2′-bipyridine ligand and by four bridging Br^−^ anions. The bridging function of the Br^−^ anions leads to a polymeric chain running along the *c* axis. Weak C—H⋯π inter­actions observed between adjacent chains are effective in the stabilization of the three-dimensional packing.

## Related literature

For related structures, see: Ahmadi *et al.* (2008 ▶); Alizadeh *et al.* (2010 ▶); Amani *et al.* (2009 ▶); Bellusci *et al.* (2008 ▶); Han *et al.* (2006 ▶); Hojjat Kashani *et al.* (2008 ▶); Kalateh *et al.* (2008 ▶, 2010 ▶); Shirvan & Haydari Dezfuli (2012 ▶); Sofetis *et al.* (2006 ▶); Willett *et al.* (2001 ▶); Yousefi *et al.* (2008 ▶); Zhang (2007 ▶).
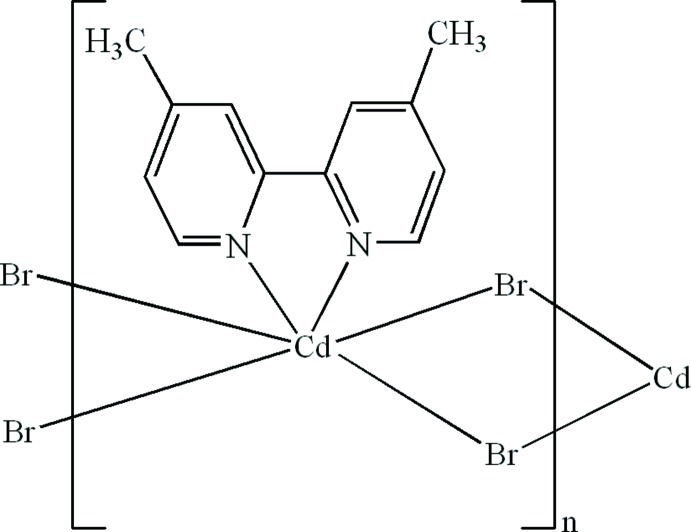


## Experimental

### 

#### Crystal data

[CdBr_2_(C_12_H_12_N_2_)]
*M* *_r_* = 456.45
Monoclinic, 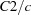

*a* = 17.979 (4) Å
*b* = 10.5319 (18) Å
*c* = 7.4496 (16) Å
β = 108.403 (17)°
*V* = 1338.5 (5) Å^3^
*Z* = 4
Mo *K*α radiation
μ = 7.58 mm^−1^
*T* = 298 K
0.25 × 0.21 × 0.20 mm

#### Data collection

Bruker APEXII CCD area-detector diffractometer
Absorption correction: multi-scan (*SADABS*; Bruker, 2001 ▶) *T*_min_ = 0.188, *T*_max_ = 0.246
3392 measured reflections
1313 independent reflections
909 reflections with *I* > 2σ(*I*)
*R*_int_ = 0.095

#### Refinement

*R*[*F* ^2^ > 2σ(*F* ^2^)] = 0.079
*wR*(*F* ^2^) = 0.155
*S* = 1.24
1313 reflections
78 parameters
H-atom parameters constrained
Δρ_max_ = 1.20 e Å^−3^
Δρ_min_ = −0.88 e Å^−3^

### 

Data collection: *APEX2* (Bruker, 2007 ▶); cell refinement: *SAINT* (Bruker, 2007 ▶); data reduction: *SAINT*; program(s) used to solve structure: *SHELXTL* (Sheldrick, 2008 ▶); program(s) used to refine structure: *SHELXTL*; molecular graphics: *SHELXTL*; software used to prepare material for publication: *SHELXTL*.

## Supplementary Material

Crystal structure: contains datablock(s) I, global. DOI: 10.1107/S1600536812046636/xu5649sup1.cif

Structure factors: contains datablock(s) I. DOI: 10.1107/S1600536812046636/xu5649Isup2.hkl

Additional supplementary materials:  crystallographic information; 3D view; checkCIF report

## Figures and Tables

**Table 1:** Selected bond lengths (Å)

Cd1—N1	2.357 (10)
Cd1—Br1	2.6852 (17)
Cd1—Br1^i^	2.8789 (16)

Symmetry code: (i) 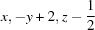.

**Table 2:** Hydrogen-bond geometry (Å, °) *Cg* is the centroid of the N1-pyridine ring.

*D*—H⋯*A*	*D*—H	H⋯*A*	*D*⋯*A*	*D*—H⋯*A*
C4—H4*B*⋯*Cg* ^ii^	0.96	2.84	3.575 (16)	135

Symmetry code: (ii) 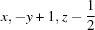.

**Fig. 1.**
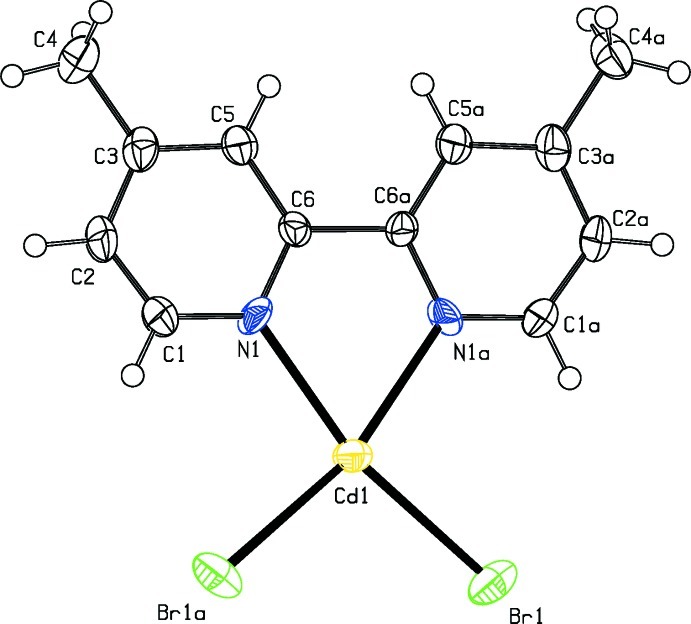
The molecular structure of the title molecule, with the atom-numbering scheme. Displacement ellipsoids are drawn at the 50% probability level.

## supplementary crystallographic information

### Comment

Recently, we reported the synthes and crystal structure of [CdBr_2_(4,4'-dmbpy)(DMSO)], (Shirvan & Haydari Dezfuli, 2012) [where 4,4'-dmbpy is 4,4'-dimethyl-2,2'-bipyridine and DMSO is dimethyl sulfoxide]. 4,4'-Dimethyl-2,2'-bipyridine is a good bidentate ligand, and numerous complexes with 4,4'-dmbipy have been prepared, such as that of mercury (Kalateh *et al.*, 2008; Yousefi *et al.*, 2008), indium (Ahmadi *et al.*, 2008), iron (Amani *et al.*, 2009), platin (Hojjat Kashani *et al.*, 2008), silver (Bellusci *et al.*, 2008), gallium (Sofetis *et al.*, 2006), copper (Willett *et al.*, 2001), cadmium (Kalateh *et al.*, 2010) and zinc (Alizadeh *et al.*, 2010). Here, we report the synthesis and structure of the title compound.

The asymmetric unit of the title compound, (Fig. 1), contains half-molecule; a twofold rotation axis passes through the Cd atom. The Cd^II^ cation is six-coordinated in a distorted octahedral geometry formed by two N atoms from the 4,4'-dimethyl-2,2'-bipyridine ligand and four bridging Br^-^ anions. The bridging function of the Br^-^ anions leads to a polymeric chain running along the *b* axis. The Cd—N and Cd—Br bond lengths and angles (Table 1) are within normal range [Cd(phen)(µ-Br)_2_]_n_, (Zhang, 2007) and [Cd(bipy)(µ-Br)_2_]_n_, (Han *et al.*, 2006) [where phen is 1,10-phenanthroline and bipy is 2,2'-bipyridine].

### Experimental

For the preparation of the title compound, a solution of 4,4'-dimethyl-2,2'-bipyridine (0.25 g, 1.33 mmol) in methanol (10 ml) was added to a solution of CdBr_2_.4H_2_O, (0.46 g, 1.33 mmol) in methanol (5 ml) at room temperature. The suitable crystals for X-ray diffraction experiment were obtained by methanol diffusion to a colorless solution in dimethylformamide. Suitable crystals were isolated after one week (yield; 0.45 g, 74.1%).

### Refinement

H atoms were positioned geometrically with C—H = 0.93–0.96 Å and constrained to ride on their parent atoms, *U*_iso_(H) = 1.2*U*_eq_(C).

### Crystal data

**Table:**

[CdBr_2_(C_12_H_12_N_2_)]	*F*(000) = 864
*M**_r_* = 456.45	*D*_x_ = 2.265 Mg m^−^^3^
Monoclinic, *C*2/*c*	Mo *K*α radiation, λ = 0.71073 Å
Hall symbol: -C 2yc	Cell parameters from 3392 reflections
*a* = 17.979 (4) Å	θ = 2.3–26.0°
*b* = 10.5319 (18) Å	µ = 7.58 mm^−^^1^
*c* = 7.4496 (16) Å	*T* = 298 K
β = 108.403 (17)°	Prism, colorless
*V* = 1338.5 (5) Å^3^	0.25 × 0.21 × 0.20 mm
*Z* = 4	

### Data collection

**Table:**

Bruker APEXII CCD area-detector diffractometer	1313 independent reflections
Radiation source: fine-focus sealed tube	909 reflections with *I* > 2σ(*I*)
Graphite monochromator	*R*_int_ = 0.095
ω scans	θ_max_ = 26.0°, θ_min_ = 2.3°
Absorption correction: multi-scan (*SADABS*; Bruker, 2001)	*h* = −22→22
*T*_min_ = 0.188, *T*_max_ = 0.246	*k* = −12→12
3392 measured reflections	*l* = −7→9

### Refinement

**Table:**

Refinement on *F*^2^	Primary atom site location: structure-invariant direct methods
Least-squares matrix: full	Secondary atom site location: difference Fourier map
*R*[*F*^2^ > 2σ(*F*^2^)] = 0.079	Hydrogen site location: inferred from neighbouring sites
*wR*(*F*^2^) = 0.155	H-atom parameters constrained
*S* = 1.24	*w* = 1/[σ^2^(*F*_o_^2^) + (0.0098*P*)^2^ + 54.7076*P*] where *P* = (*F*_o_^2^ + 2*F*_c_^2^)/3
1313 reflections	(Δ/σ)_max_ = 0.022
78 parameters	Δρ_max_ = 1.20 e Å^−^^3^
0 restraints	Δρ_min_ = −0.88 e Å^−^^3^

### Special details

**Table:**

Geometry. All e.s.d.'s (except the e.s.d. in the dihedral angle between two l.s. planes) are estimated using the full covariance matrix. The cell e.s.d.'s are taken into account individually in the estimation of e.s.d.'s in distances, angles and torsion angles; correlations between e.s.d.'s in cell parameters are only used when they are defined by crystal symmetry. An approximate (isotropic) treatment of cell e.s.d.'s is used for estimating e.s.d.'s involving l.s. planes.
Refinement. Refinement of *F*^2^ against ALL reflections. The weighted *R*-factor *wR* and goodness of fit *S* are based on *F*^2^, conventional *R*-factors *R* are based on *F*, with *F* set to zero for negative *F*^2^. The threshold expression of *F*^2^ > σ(*F*^2^) is used only for calculating *R*-factors(gt) *etc*. and is not relevant to the choice of reflections for refinement. *R*-factors based on *F*^2^ are statistically about twice as large as those based on *F*, and *R*- factors based on ALL data will be even larger.

### Fractional atomic coordinates and isotropic or equivalent isotropic displacement parameters (Å^2^)

**Table:**

	*x*	*y*	*z*	*U*_iso_*/*U*_eq_	
Cd1	0.0000	0.92501 (14)	0.2500	0.0312 (4)	
Br1	0.09337 (9)	1.08683 (16)	0.4997 (2)	0.0439 (5)	
N1	−0.0688 (6)	0.7414 (11)	0.1090 (15)	0.030 (3)	
C6	−0.0397 (7)	0.6266 (12)	0.1779 (18)	0.023 (3)	
C5	−0.0804 (7)	0.5157 (13)	0.111 (2)	0.030 (3)	
H5	−0.0585	0.4376	0.1574	0.036*	
C1	−0.1384 (8)	0.7427 (15)	−0.025 (2)	0.042 (4)	
H1	−0.1579	0.8209	−0.0767	0.050*	
C3	−0.1535 (8)	0.5209 (14)	−0.025 (2)	0.031 (3)	
C4	−0.2002 (8)	0.4012 (14)	−0.094 (2)	0.040 (4)	
H4A	−0.2098	0.3586	0.0101	0.048*	
H4B	−0.1712	0.3462	−0.1505	0.048*	
H4C	−0.2493	0.4229	−0.1869	0.048*	
C2	−0.1825 (8)	0.6396 (14)	−0.091 (2)	0.036 (3)	
H2	−0.2318	0.6478	−0.1806	0.043*	

### Atomic displacement parameters (Å^2^)

**Table:**

	*U*^11^	*U*^22^	*U*^33^	*U*^12^	*U*^13^	*U*^23^
Cd1	0.0363 (8)	0.0203 (7)	0.0304 (9)	0.000	0.0011 (6)	0.000
Br1	0.0434 (10)	0.0394 (9)	0.0520 (12)	−0.0169 (7)	0.0195 (8)	−0.0153 (8)
N1	0.032 (6)	0.027 (5)	0.022 (6)	−0.009 (5)	−0.005 (4)	−0.006 (5)
C6	0.018 (6)	0.024 (6)	0.030 (7)	0.002 (5)	0.009 (5)	0.002 (6)
C5	0.019 (6)	0.031 (7)	0.045 (9)	0.002 (5)	0.015 (6)	−0.002 (6)
C1	0.034 (8)	0.030 (7)	0.046 (9)	0.003 (6)	−0.008 (7)	0.010 (7)
C3	0.027 (7)	0.042 (8)	0.023 (7)	0.001 (6)	0.005 (5)	−0.010 (6)
C4	0.037 (8)	0.044 (9)	0.035 (8)	−0.012 (7)	0.006 (6)	−0.003 (7)
C2	0.026 (7)	0.042 (8)	0.031 (8)	0.002 (6)	−0.005 (6)	0.004 (7)

### Geometric parameters (Å, º)

**Table:**

Cd1—N1^i^	2.357 (10)	C5—C3	1.383 (18)
Cd1—N1	2.357 (10)	C5—H5	0.9300
Cd1—Br1^i^	2.6852 (17)	C1—C2	1.34 (2)
Cd1—Br1	2.6852 (17)	C1—H1	0.9300
Cd1—Br1^ii^	2.8789 (16)	C3—C2	1.39 (2)
Cd1—Br1^iii^	2.8790 (16)	C3—C4	1.513 (19)
Br1—Cd1^iii^	2.8789 (16)	C4—H4A	0.9600
N1—C1	1.331 (16)	C4—H4B	0.9600
N1—C6	1.353 (17)	C4—H4C	0.9600
C6—C5	1.384 (18)	C2—H2	0.9300
C6—C6^i^	1.49 (2)		
			
N1^i^—Cd1—N1	69.7 (5)	N1—C6—C6^i^	116.2 (7)
N1^i^—Cd1—Br1^i^	162.8 (3)	C5—C6—C6^i^	122.4 (7)
N1—Cd1—Br1^i^	95.0 (3)	C3—C5—C6	120.1 (13)
N1^i^—Cd1—Br1	95.0 (3)	C3—C5—H5	119.9
N1—Cd1—Br1	162.8 (3)	C6—C5—H5	119.9
Br1^i^—Cd1—Br1	101.21 (9)	N1—C1—C2	125.0 (14)
N1^i^—Cd1—Br1^ii^	85.5 (3)	N1—C1—H1	117.5
N1—Cd1—Br1^ii^	90.4 (3)	C2—C1—H1	117.5
Br1^i^—Cd1—Br1^ii^	86.77 (5)	C5—C3—C2	117.4 (13)
Br1—Cd1—Br1^ii^	96.39 (5)	C5—C3—C4	121.0 (13)
N1^i^—Cd1—Br1^iii^	90.4 (3)	C2—C3—C4	121.6 (12)
N1—Cd1—Br1^iii^	85.5 (3)	C3—C4—H4A	109.5
Br1^i^—Cd1—Br1^iii^	96.39 (5)	C3—C4—H4B	109.5
Br1—Cd1—Br1^iii^	86.77 (5)	H4A—C4—H4B	109.5
Br1^ii^—Cd1—Br1^iii^	175.03 (9)	C3—C4—H4C	109.5
Cd1—Br1—Cd1^iii^	93.23 (5)	H4A—C4—H4C	109.5
C1—N1—C6	116.9 (12)	H4B—C4—H4C	109.5
C1—N1—Cd1	124.3 (10)	C1—C2—C3	119.1 (12)
C6—N1—Cd1	118.6 (8)	C1—C2—H2	120.4
N1—C6—C5	121.3 (11)	C3—C2—H2	120.4
			
N1^i^—Cd1—Br1—Cd1^iii^	−90.1 (3)	Br1^iii^—Cd1—N1—C6	−89.3 (10)
N1—Cd1—Br1—Cd1^iii^	−63.5 (10)	C1—N1—C6—C5	0 (2)
Br1^i^—Cd1—Br1—Cd1^iii^	95.88 (5)	Cd1—N1—C6—C5	175.0 (10)
Br1^ii^—Cd1—Br1—Cd1^iii^	−176.16 (7)	C1—N1—C6—C6^i^	177.6 (15)
Br1^iii^—Cd1—Br1—Cd1^iii^	0.0	Cd1—N1—C6—C6^i^	−7.7 (19)
N1^i^—Cd1—N1—C1	177.1 (15)	N1—C6—C5—C3	−2 (2)
Br1^i^—Cd1—N1—C1	−11.0 (12)	C6^i^—C6—C5—C3	−179.3 (14)
Br1—Cd1—N1—C1	148.7 (10)	C6—N1—C1—C2	2 (2)
Br1^ii^—Cd1—N1—C1	−97.8 (12)	Cd1—N1—C1—C2	−171.9 (13)
Br1^iii^—Cd1—N1—C1	85.0 (12)	C6—C5—C3—C2	1 (2)
N1^i^—Cd1—N1—C6	2.9 (7)	C6—C5—C3—C4	−177.8 (13)
Br1^i^—Cd1—N1—C6	174.7 (9)	N1—C1—C2—C3	−3 (3)
Br1—Cd1—N1—C6	−25.6 (17)	C5—C3—C2—C1	1 (2)
Br1^ii^—Cd1—N1—C6	87.9 (10)	C4—C3—C2—C1	−179.7 (15)

Symmetry codes: (i) −*x*, *y*, −*z*+1/2; (ii) *x*, −*y*+2, *z*−1/2; (iii) −*x*, −*y*+2, −*z*+1.

### Hydrogen-bond geometry (Å, º)

Cg is the centroid of the N1-pyridine ring.

**Table:**

*D*—H···*A*	*D*—H	H···*A*	*D*···*A*	*D*—H···*A*
C4—H4*B*···*Cg*^iv^	0.96	2.84	3.575 (16)	135

Symmetry code: (iv) *x*, −*y*+1, *z*−1/2.
